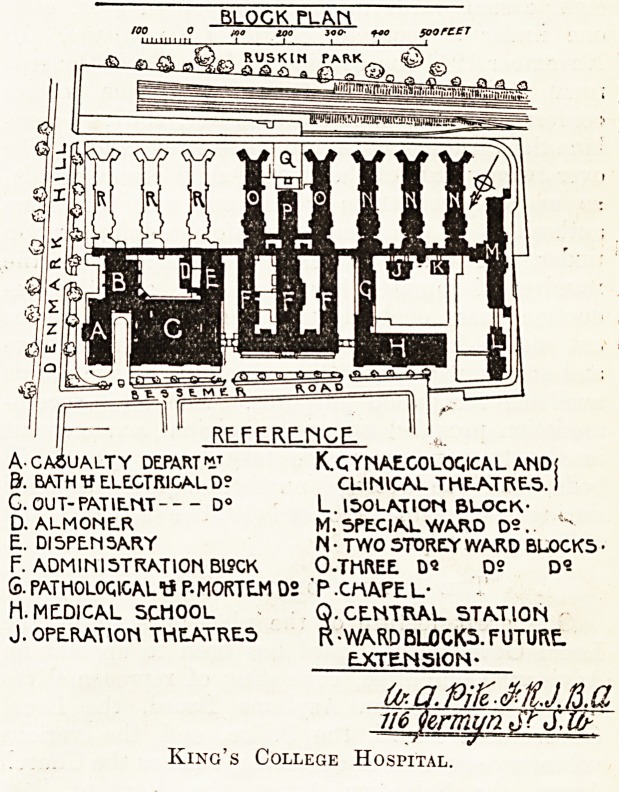# Infirmary v. Hospital Architecture

**Published:** 1913-04-05

**Authors:** A. Saxon Snell


### INFIRMARY v. HOSPITAL ARCHITECTURE.

By A. SAXON SNELL, F.R.I.B.A., F.R.San.Inst.

To make any sort of comparison between infirmaries and hospitals it is necessary in the first place to consider the origin and purpose of each. For the most part, the former have been in England county hospitals with less than 200 beds and the latter great hospitals with considerably more accommodation. Infirmary is generally the name given to all the chief hospitals in Scotland. Both hospitals and infirmaries were originally constituted as private establishments, founded in the first place by the benefactions of humane and generous men and women for the care and treatment of those sick and injured persons who were too poor to command the services of skilled physicians, surgeons, and trained nurses. In some cases they have received from time to time grants of money and land, etc., from public and royal funds. They were and are maintained by free-will offerings in the way of annual subscriptions, donations, and legacies. They are vested in trustees and managed by committees elected generally by certain classes of subscribers. The patients treated were drawn mostly from the lower and working classes, and not necessarily from the neighbourhood of the particular hospital. They might come from any district on the recommendation of subscribers and others. Accident cases are admitted and treated in the casualty department, and, if necessary, taken into the hospital and cared for until convalescent. The patient's title to admission and treatment is solely that of necessity and urgency.

Infirmary wards have for decades of years been adjuncts to the workhouses for the housing and treatment of sick inmates. They were, in fact, part of the workhouse buildings and under the superintendence of the master. This, indeed, obtains to the present day in the smaller institutions. Gathorne Hardy's Act, 1867, created the modern Poor-Law infirmary, and with the advancement of medicine and improvement of nursing, the control of infirmaries passed from the workhouse master to that of a medical superintendent, with trained and paid nurses in place of untrained women selected from the workhouse inmates.

In large and urban workhouses, which have been in most cases rebuilt, the infirmary has been made a separate institution, and as often as not it is placed at a distance from the workhouse, and, may be, in a different parish—in some cases in the open country; technically it is still an adjunct of, and its patients are drawn from, the workhouse. Of late years patients are also sent direct from their homes by the order of out-relief or dispensary committees. It is obvious that a number of these patients will be suffering only from diseases, or weakness connected with old age, and a low standard of general health. Such diseases in the non-pauper class are treated by the general practitioner in the patients' own homes. They are drafted into hospitals only for special treatment or surgical operations, which could be carried out at their own homes only at great inconvenience and prohibitive cost. In the hospitals the medical and surgical work and the nursing must be greater and more varied than in an infirmary, and they entail the services of a far larger medical and nursing staff. Compare, for instance, the proportion of nurses to patients, which in an infirmary is about 1 to every 9, and in a hospital 1 to every $2\frac{1}{2}$.

In the matter of nursing in infirmaries I should add that an enormous improvement has been made, mainly, I think, through the work of the late Miss Twining and Miss Wilson. The education and treatment are approximately similar to that of hospital nurses, but necessarily the training and experience gained are not so thorough. Hospital nurses, too, are recruited from a higher class of women. Dispensaries, which correspond to the out-patients' departments of hospitals, are maintained within the confines of each parish, though not necessarily as part of, or contiguous to, the workhouse.

Willesden Parish Infirmary.

The removal of an urban hospital to more open surroundings has been almost impracticable until quite recent years. Apart from the question of cost, there was that of accessibility to the great physicians and surgeons upon whose gratuitous services the hospitals depend so largely. The convenience of the motor-car has lessened that particular difficulty.

So far, only one large London hospital has migrated to the more open spaces of the suburbs; and the journey from Harley Street to Denmark Hill by motor-car or taxicab is certainly not longer than it used to be by brougham or "hansom" to the old hospital. The removal of King's College Hospital is a great and bold undertaking, and in the right direction. It is said that Westminster Hospital will soon follow suit.

A large modern infirmary is administered by a medical superintendent, with the assistance of a steward or storekeeper and a matron. In the medical work he is assisted by one or more qualified assistants. Of course, the general control is under the direction of a committee of the Guardians. In a hospital the purely administrative work is conducted by a secretary and his staff, including a steward and matron. For the care and treatment of the patients there are house physicians and surgeons elected for a short term of years and at comparatively small remuneration; the great opportunities for gaining practice and "kudos" are compensations.

In comparing the efficiency of the medical and surgical service as between infirmaries and hospitals, it may be taken that the qualification of the infirmary doctor will generally be higher than that of the hospital house surgeon; but as a set-off to any inferiority in this respect the latter is assisted by a band of the most highly qualified practising physicians and surgeons (mostly great specialists), who give their services, and whose skill is placed freely at the call of the poorest patient. This is the special and peculiar feature of hospital treatment, and its value is incalculable. Would such services be given in the same way if the hospitals were taken over by the State?

With regard to the design and arrangement of hospitals and infirmaries, we are in a better position for making comparisons; and it is in this matter that, as a general rule, the former must give

Charing Cross Hospital.

place to the latter. Many years ago, in reply to some disparaging remarks anent infirmaries I retorted with regrettable impatience that as compared with hospitals, they "cost about half, and were twice as efficient from a hygienic standpoint." Although I am not prepared at the present day to maintain this proposition in its crude exaggeration, it cannot be denied that it still holds in a much less degree, and the reasons are not far to seek.

In your issue of March 8, 1913, a correspondent ("One Who Knows") sets forth, under the heading "Wanted, a Hospital Architect," an indictment of architects whom he assumes are responsible for the mistakes which sometimes are but too evident in hospital buildings; and he quotes some obscure phrases from an article which (he says), "judging from the technical words used, would seem to have come from an architectural source." Now, although I am not prepared to deny that architects —especially those without special knowledge of the subject—have been responsible to some extent for poverty of design, and unnecessary cost in hospital buildings, "One Who [thinks he] Knows" must look a little further for the defects of which he complains. The promoters of a new hospital, for instance, do not, as a rule, and as a first step, take the architect into their counsels in the selection of a site. The general rule is to acquire a site upon the advice of anyone but the architect, and for any reason other than its suitability from the point of view of hygiene, and thereafter to ask an architect —or several—to show how it can be adapted for the hospital required. It is usually far too small. No doubt the medical members of the committee are consulted in the matter (though not always). Even so, it must be borne in mind that as a general rule their training—wide and thorough as it is—includes the laws of hygiene only in a limited sense; and this will continue until the course of instruction leading to the D.P.H. degree is compulsory.

The selection of a site is a matter of the very first importance, and few people seem to grasp the necessity of sufficient area. Who, for instance, was responsible for the selection of the sites of two of the finest and newest of our hospitals—*i.e.*, King's College and Manchester Royal? Certainly not the architects. Neither is of adequate area for anything like ideal arrangement of the buildings, and to secure a real sufficiency of light and air to the ward blocks.

In the arrangement of his building, the architect may be largely influenced by these same medical advisers and others with ideas of their own. That is right in every way, for a good hospital can be designed only with the help of the competent physicians and surgeons who know the conditions of their work so much more intimately than an architect, however skilled he may be. On the other hand, he (the architect) may—unless he is a very strong man—be over-ridden in matters with which he is better qualified to deal. In cases of disagreement he has little chance to enforce his views, for more reasons than I have space or temerity to advance; and there is no appeal to a higher authority. I am not forgetting the committee of King Edward's Hospital Fund; but its powers are, of course, limited, and—I add in all humility—I doubt whether it has as yet a settled standard to refer to, or whether an architect is included among its Commissioners. Now, in the case of infirmaries there is the great difference of the guiding hand of the Local Government Board.

The arrangement and construction of all Poor-Law buildings in this country is subject to the criticism and control of this Board, which has always had in its service men with high expert knowledge of the subject, and the means of collecting the experience and knowledge of savants, whose teachings have revolutionised our ideas of hygiene and sanitation. It is, and always has been, the guardian of public health; and has fostered and enforced the laws which have done so much to improve the health of the community. It also controls the expenditure upon all public buildings for the housing or treatment of the sick poor; and in this way the comparative ignorance and possible extravagance of local bodies are corrected or kept in check. With such advisers as Sir Arthur Downes and other medical inspectors, Mr. Brook Kitchin, the architect, and his predecessor, the late P. Gordon Smith, the control is and has been very efficient.

One of the points which is specially insisted upon by the Local Government Board is a site of adequate area, to permit of the free circulation of air in and around the ward blocks. Avoidance of connecting corridors above the level of the ground floor, and of closed quadrangles are others; and I think it is safe to conjecture that (for instance) neither the site nor the crowded buildings of the new King's College Hospital would have been passed by its advisers.

The Willesden Parish Infirmary, of which I show a block plan, gives some idea of the area and arrangement generally approved. The total number of beds will be 800, and the area is just over eleven acres—allowing one acre to seventy-three patients; and that is by no means too large. It was intended originally for 600 only. For a hospital with its great preponderance of administrative buildings, out-patients' and casualty departments, medical school, etc., twice that area would not be extravagant. The site of King's College is about twelve acres for 600 beds, or one to fifty patients. At the Hamburg (Eppendorf) Hospital the acreage is one to thirty-seven patients; at Berlin (Friedrichshain) one to thirty-two; Charlottenburg one to thirty-seven; Heidelberg one to forty. In France there is St. Denis, with one to twenty-six; Montpelier, one to twenty-seven. The Johns Hopkins, at Baltimore (U.S.A.), has one to twenty-six. Some, at least, of these are in urban districts. In but few cases is land so costly as to be really prohibitive, and it may form but a small proportion of the total expense. I endeavoured to prove this in a paper read before the Royal Institute of British Architects in February last.*

In rebuilding or adding to old hospitals on the original sites, it may be difficult or disproportionately costly to acquire really adequate area, but these cases are exceptional. The block plan which I give of Charing Cross Hospital shows a very crowded site indeed; but it will be noted that the best positions are devoted to the ward blocks. In this case I know that the governors earnestly desired to incorporate the site of the adjoining Westminster Ophthalmic Hospital, which would have made an island site of the whole, and have vastly improved the buildings.

In view of the greater space required for administrative and other purposes in hospitals, it is obvious that they must of necessity cost more per bed than infirmaries; but in addition to this, it must be acknowledged that the former are generally more lavishly finished than the latter; not always, if at all, in a spirit of extravagance, but often by the will of those who provide the money.

An eminent surgeon once told me that he could carry an operation through as successfully in a well-lighted white-washed room as in the most elaborate and marble-lined operating theatre; "but," he added in effect, "the rich man who provides the money thinks otherwise, and he would sooner pay a heavy price for the fine finishings and fittings which he believes essential. He is guided by his experience of the comfort and efficiency of his own costly mansion."

It is not to be denied, too, that in the details of fittings and finishings infirmaries have been inferior to hospitals until quite recent times. Indeed, I think it safe to say that in these matters hospitals have led the way for many years, and they are often of vital importance.

I have not the space to draw attention to many other matters of comparison between these different institutions. In conclusion, I may add, then, when,

King's College Hospital.

if ever, the State takes over the hospitals it will be no small undertaking. There are those who, thinking of the large invested funds and properties attached, imagine that the cost to the community will be small, while the future maintenance will be more efficient and less costly. If they base their calculations upon the cost and upkeep of Poor-Law infirmaries they may be quickly undeceived, if only because the work of a general hospital is so much more varied and extensive, and so large a proportion of the service is given without fee.

* *Journal of the R.I.B.A.*, Third Series, Vol. xx., No. 9.

## Figures and Tables

**Figure f1:**
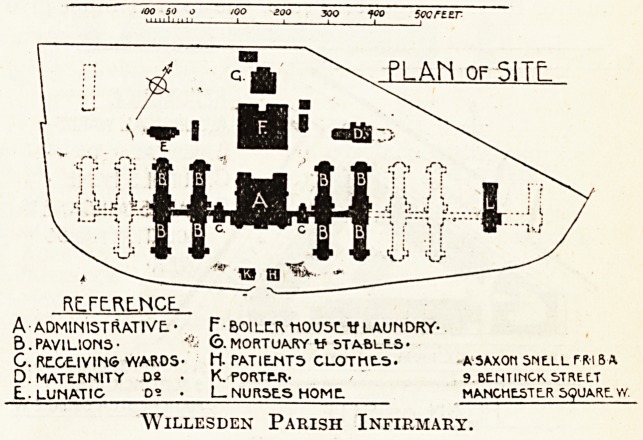


**Figure f2:**
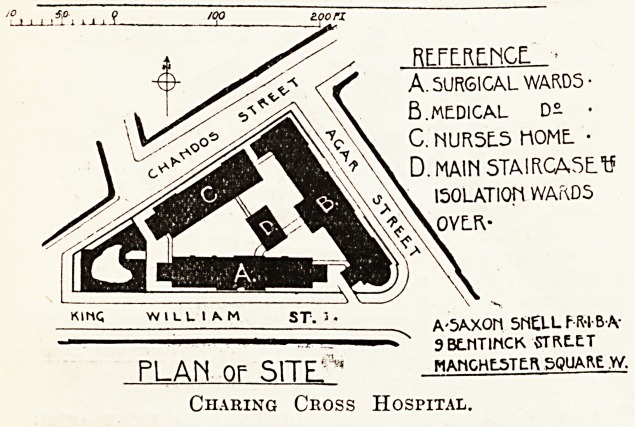


**Figure f3:**